# Hydrogen Peroxide Response in Leaves of Poplar (*Populus simonii × Populus nigra*) Revealed from Physiological and Proteomic Analyses

**DOI:** 10.3390/ijms18102085

**Published:** 2017-10-02

**Authors:** Juanjuan Yu, Xin Jin, Xiaomei Sun, Tianxiang Gao, Xiaomei Chen, Yimin She, Tingbo Jiang, Sixue Chen, Shaojun Dai

**Affiliations:** 1 Alkali Soil Natural Environmental Science Center, Northeast Forestry University, Key Laboratory of Saline-alkali Vegetation Ecology Restoration, Ministry of Education, Harbin 150040, China; yujuan8186@163.com (J.Y.); JinXin721612@163.com (X.J.); tbjiang@yahoo.com (T.J.); 2 Development Centre of Plant Germplasm Resources, College of Life and Environmental Sciences, Shanghai Normal University, Shanghai 200234, China; meimeixpz@163.com (X.S.); thalia1@163.com (T.G.); schen@ufl.edu (S.C.); 3 Shanghai Center for Plant Stress Biology, Chinese Academy of Sciences, Shanghai 201602, China; chenxm@sibs.ac.cn (X.C.); ymshe@sibs.ac.cn (Y.S.); 4 Department of Biology, Genetics Institute, Plant Molecular and Cellular Program, Interdisciplinary Center for Biotechnology Research, University of Florida, Gainesville, FL 32610, USA

**Keywords:** *Populus simonii × Populus nigra*, leaves, H_2_O_2_ stress, proteomics

## Abstract

Hydrogen peroxide (H_2_O_2_) is one of the most abundant reactive oxygen species (ROS), which plays dual roles as a toxic byproduct of cell metabolism and a regulatory signal molecule in plant development and stress response. *Populus simonii* × *Populus nigra* is an important cultivated forest species with resistance to cold, drought, insect and disease, and also a key model plant for forest genetic engineering. In this study, H_2_O_2_ response in *P. simonii × P. nigra* leaves was investigated using physiological and proteomics approaches. The seedlings of 50-day-old *P. simonii × P. nigra* under H_2_O_2_ stress exhibited stressful phenotypes, such as increase of in vivo H_2_O_2_ content, decrease of photosynthetic rate, elevated osmolytes, antioxidant accumulation, as well as increased activities of several ROS scavenging enzymes. Besides, 81 H_2_O_2_-responsive proteins were identified in the poplar leaves. The diverse abundant patterns of these proteins highlight the H_2_O_2_-responsive pathways in leaves, including 14-3-3 protein and nucleoside diphosphate kinase (NDPK)-mediated signaling, modulation of thylakoid membrane structure, enhancement of various ROS scavenging pathways, decrease of photosynthesis, dynamics of proteins conformation, and changes in carbohydrate and other metabolisms. This study provides valuable information for understanding H_2_O_2_-responsive mechanisms in leaves of *P. simonii* × *P. nigra*.

## 1. Introduction

Various environmental stresses usually affect reactive oxygen species (ROS) homeostasis in plants, leading to the generation of excess ROS, such as singlet oxygen (^1^O_2_), superoxide anion radicals (O_2_^•−^), hydrogen peroxide (H_2_O_2_), and hydroxyl radicals (HO^•^). Among them, H_2_O_2_ is the most abundant ROS in plant cells during photosynthesis, photorespiration, and respiration processes [1]. The relatively stable non-radical H_2_O_2_ can easily penetrate membrane through water channels, functioning as a likely long-distance signaling molecule in plant growth and stress perception [2]. Moreover, H_2_O_2_ has been proven to be a regulator of many physiological processes, such as cell wall modulation, senescence, phytoalexin production, photosynthesis, stomatal movement, and cell cycle [3]. Excess H_2_O_2_ has obvious oxidative destruction of diverse molecules (e.g., proteins, nucleic acids, carbohydrates, and unsaturated lipids) in plant cells [4], which disturbs cellular activity and causes programmed cell death [2]. Interestingly, considerable investigations have shown that low concentration of H_2_O_2_ can improve seed germination [5] and plant resistance to various abiotic and biotic stresses, such as salinity [6,7], osmotic stress [8], aluminum [9], heat [6], chilling [10], paraquat [11], and potato virus Y infection [12]. H_2_O_2_ changes in cells showed as a bell-shaped response with an optimum, depending on the plant species, developmental stages, cell types, and environmental conditions [2,7].

Previous transcriptomic analyses have reported a large number of H_2_O_2_-responsive genes in various plants. For example, more than 170 non-redundant expressed sequence tags (ESTs) in Arabidopsis [13], 713 ESTs in catalase (CAT)-deficient tobacco plants [14], and 437 transcripts in CAT-deficient Arabidopsis [15] were identified as H_2_O_2_ responsive genes. In addition, 6156, 6875 and 3276 transcripts were differentially expressed in three wheat lines including a powdery mildew resistant (PmA) line and two susceptible (Han and Cha) lines [16]. Recently, more than 385 H_2_O_2_-responsive proteins were identified in leaves from rice (*Oryza sativa* L. cv. 93–11) [17], citrus (*Citrus aurantium* L.) [18], *Brachypodium distachyon* [19], and wheat (*Triticum aestivum* L.) [20] using two-dimensional gel electrophoresis (2DE)-based or isobaric tags for relative and absolute quantification (iTRAQ)-based proteomics approaches. The dynamic abundance patterns of these proteins imply that ROS homeostasis, signaling, photosynthesis, energy metabolism, lipid metabolism, and protein turnover play important roles in leaf H_2_O_2_ response. However, most of these proteomics studies focused on model plants and crops. Proteomic analysis of the response of forest trees to H_2_O_2_ stress has not been reported.

Poplar trees are widely planted, and poplar woods are commonly used for building materials, furniture and paper [21]. With the sequencing of *Populus* genome, poplar has emerged as a model system for molecular and genetic studies of forest trees [21]. *Populus simonii × Populus nigra*, also called as *Populus xiaohei*, is the hybrid of *Populus simonii* and *Populus nigra*, which widely distributes in northern China, and has been well used for afforestation and commercial forest. *P. simonii × P. nigra* is a fast-growing tree species with excellent properties of resistance to cold, drought, insect and disease. The early studies of *P. simonii* × *P. nigra* were mainly focused on cultivation and germplasm introduction [22]. Recently, research has been focused on using transgenic technology to improve salt and drought tolerance [23], insect resistance [24], and disease resistance [25]. Besides, transcriptics and proteomics have been utilized to study *P. simonii × P. nigra* [26,27,28,29]. Thousands of genes were shown to be differentially expressed in response to NaCl stress using cDNA-amplified fragment length polymorphism approach [26] and Solexa/illumine digital gene expression technique [27]. Additionally, genome-wide and proteomic analysis of a TaLEA (*Tamarix androssowii* late embryogenesis abundant gene)-introduced transgenic *P. simonii* × *P. nigra* dwarf mutant showed 537 genes and 99 proteins were significantly altered, respectively [28,29]. However, there is still lack of proteomics information of *P. simonii × P. nigra* in response to stresses. Thus, the dynamic proteomic analysis of *P. simonii × P. nigra* under H_2_O_2_ stress is important for further investigation of the molecular mechanism of oxidative stress and biotechnological manipulation with the aim of enhancing poplar stress tolerance.

In this study, we performed physiological and proteomic analyses of *P. simonii × P. nigra* leaves under 0, 12, 24 and 36 mM H_2_O_2_ treatments for 6 h. Our results indicate that modulation of thylakoid structure, ROS scavenging pathways, photosynthesis, and protein conformation play critical roles in *P. simonii × P. nigra* leaves in response to H_2_O_2_. These results provide new insights into the molecular mechanisms underlying poplar response to H_2_O_2_ stress.

## 2. Results

### 2.1. Photosynthesis under Hydrogen Peroxide (H_2_O_2_) Stress

**Figure 1:**
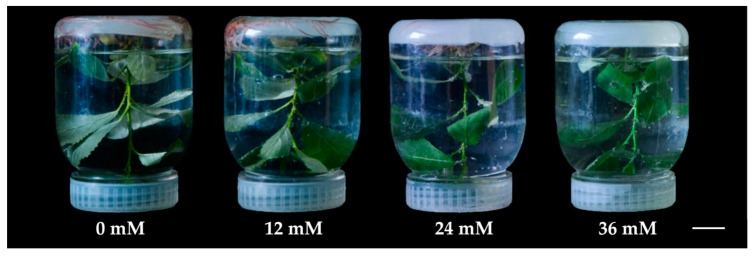
The morphology changes of *Populus simonii × Populus nigra* under hydrogen peroxide (H_2_O_2_) stress. The aerial portion of 50-day-old seedling of *P. simonii × P. nigra* was immersed in 0, 12, 24 and 36 mM H_2_O_2_ for 6 h, respectively. Bar = 0.2 cm.

**Figure 2:**
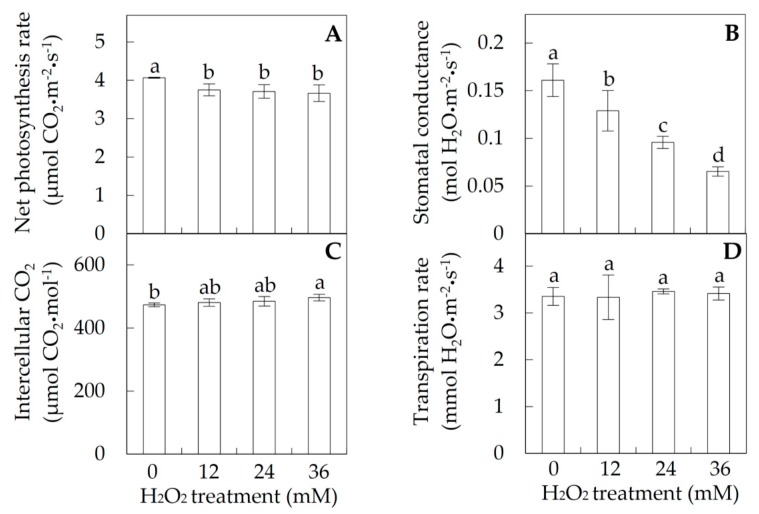
Photosynthetic characteristics of *Populus simonii × Populus nigra* leaves under H_2_O_2_ treatment: (**A**) photosynthesis rate (Pn); (**B**) stomata conductance (Gs); (**C**) intercellular CO_2_ (Ci); and (**D**) transpiration rate (Tr). The values were determined after plants were treated with 0, 12, 24 and 36 mM H_2_O_2_, and were presented as means ± standard deviation (SD) (*n* = 3). The different small letters indicate significant difference (*p* < 0.05) among different treatments.

The leaves of *P. simonii × P. nigra* were immersed in 0, 12, 24 and 36 mM H_2_O_2_ solutions for 6 h, respectively (Figure 1). The photosynthetic parameters were measured to evaluate the photosynthetic changes in response to the H_2_O_2_ stress. The net photosynthetic rate (Pn) decreased from 4 μmol CO_2_∙m^−2^·s^−1^ in control to about 3.7 μmol CO_2_∙m^−2^·s^−1^ under each H_2_O_2_ treatment (Figure 2A). The stomatal conductances (Gs) under 12, 24, and 36 mM H_2_O_2_ were also reduced 1.2-, 1.7-, and 2.4-fold, respectively, when compared with control (Figure 2B). In addition, the intercellular CO_2_ concentration (Ci) increased slightly from 473.2 μmol CO_2_·mol^−1^ in control to 496.3 μmol CO_2_·mol^−1^ under 36 mM H_2_O_2_, but the transpiration rate (Tr) was not significantly altered under the H_2_O_2_ stress (Figure 2C,D).

### 2.2. Membrane Integrity and Osmolyte Accumulation in Leaves

**Figure 3:**
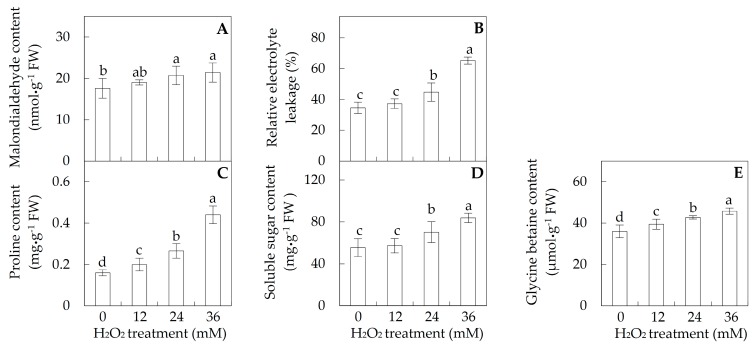
Membrane integrity and osmolyte accumulation in *Populus simonii × Populus nigra* leaves under H_2_O_2_ treatment: (**A**) malondialdehyde content; (**B**) relative electrolyte leakage; (**C**) proline content; (**D**) soluble sugar content; and (**E**) glycine betanine content. The values were determined after the plants were treated with 0, 12, 24 and 36 mM H_2_O_2_, and were presented as means ± SD (*n* = 3). The different small letters indicate significant difference (*p* < 0.05) among different treatments.

To evaluate the effects of H_2_O_2_ on membrane stability, the malondialdehyde (MDA) content and relative electrolyte leakage (REL) in leaves were determined. MDA contents and RELs were not changed under 12 mM H_2_O_2_, but increased under 24 and 36 mM H_2_O_2_ (Figure 3A,B). MDA contents were increased from 17.6 nmol·g^−1^ fresh weight (FW) in control to about 21.4 nmol·g^−1^ FW under 24 and 36 mM H_2_O_2_ (Figure 3A). For REL, a 1.3-fold increase under 24 mM H_2_O_2_ and a 1.9-fold under 36 mM H_2_O_2_ were observed when compared with control (Figure 3B).

In addition, the contents of proline and glycine betaine were increased gradually and significantly with the increasing concentration of H_2_O_2_. The proline contents under H_2_O_2_ treatment of 12, 24 and 36 mM were increased 1.3-, 1.7-, and 2.8-fold, respectively (Figure 3C). The contents of glycine betaine under three H_2_O_2_ treatments were increased 1.1-, 1.2-, 1.3-fold, respectively (Figure 3E). In addition, the contents of soluble sugar were increased 1.3-fold under 24 mM and 1.5-fold under 36 mM H_2_O_2_ (Figure 3D).

### 2.3. ROS and Antioxidant Substances Content, and Antioxidant Enzyme Activities

**Figure 4:**
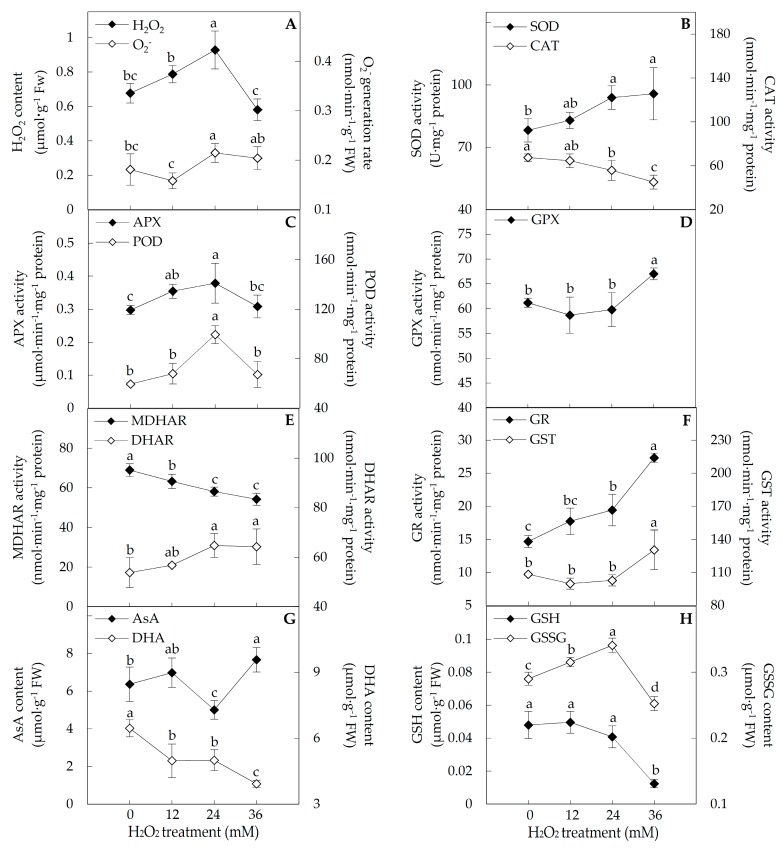
Activities of antioxidant enzymes and antioxidant contents in *Populus simonii × Populus nigra* leaves under H_2_O_2_ treatment: (**A**) H_2_O_2_ content and O_2_^•−^ generation rate; (**B**) activities of superoxide dismutase (SOD) and catalase (CAT); (**C**) activities of ascorbate peroxidase (APX) and peroxidase (POD); (**D**) glutathione peroxidase (GPX) activity; (**E**) activities of monodehydroascorbate reductase (MDHAR) and dehydroascorbate reductase (DHAR); (**F**) activities of glutathione reductase (GR) and glutathione S-transferase (GST); (**G**) contents of ascorbate (AsA) and dehydroascorbate (DHA); and (**H**) contents of reduced glutathione (GSH) content and oxidized glutathione (GSSG) content. The values were determined after plants were treated with 0, 12, 24 and 36 mM H_2_O_2_, and were presented as means ± SD (*n* = 3). The different small letters indicate significant difference (*p* < 0.05) among different treatments.

To evaluate the ROS homeostasis in the H_2_O_2_-treated leaves, the O_2_^•−^ generation rate, H_2_O_2_ content, and the activities of several ROS scavenging enzymes were analyzed. The O_2_^•−^ generation rate remained constant under 12 and 36 mM H_2_O_2_ treatments, but was increased 1.2-fold under 24 mM H_2_O_2_ treatment (Figure 4A). H_2_O_2_ content in leaves was increased 1.4-fold under 24 mM H_2_O_2_ treatment (Figure 4A). Superoxide dismutase (SOD) activity was increased about 1.2-fold under 24 and 36 mM H_2_O_2_ treatments (Figure 4B). Besides, the activities of several ROS scavenging enzymes were altered in leaves under certain H_2_O_2_ concentrations. The CAT activities were decreased 1.2-fold under 24 mM and 1.5-fold under 36 mM H_2_O_2_ treatments (Figure 4B). However, the activities of ascorbate peroxidase (APX) were increased about 1.2-fold under 12 and 24 mM H_2_O_2_, and peroxidase (POD) activity was increased 1.7-fold under 24 mM H_2_O_2_ (Figure 4C). The glutathione peroxidase (GPX) activity was increased 1.1-fold under 36 mM H_2_O_2_ treatment (Figure 4D). Moreover, the activities of three enzymes involved in the regeneration of the reduced antioxidants were all altered under the H_2_O_2_ stress. The activity of monodehydroascorbate reductase (MDHAR) was inhibited, while the activities of dehydroascorbate reductase (DHAR) and glutathione reductase (GR) were significantly increased under H_2_O_2_ treatments (Figure 4E,F). The activities of MDHAR under three H_2_O_2_ treatments were reduced 1.1-, 1.2-, and 1.3-fold, respectively. DHAR activities were increased about 1.2-fold under 24 and 36 mM H_2_O_2_, and GR activities were increased 1.3-fold under 24 mM H_2_O_2_ and 1.9-fold under 36 mM H_2_O_2_ (Figure 4E,F). In addition, the glutathione *S*-transferase (GST) activity was induced 1.2-fold under 36 mM H_2_O_2_ treatment (Figure 4F).

In addition, ascorbate (AsA), dehydroascorbate (DHA), reduced glutathione (GSH), and oxidized glutathione (GSSG) were detected in leaves in response to the H_2_O_2_ treatment. The contents of AsA were decreased 1.3-fold under 24 mM H_2_O_2_, but increased 1.2-fold under 36 mM H_2_O_2_ treatment (Figure 4G). The contents of DHA and GSH were all reduced under H_2_O_2_ treatment. DHA contents were decreased about 1.3-fold under 12 and 24 mM H_2_O_2_, and 1.6-fold under 36 mM H_2_O_2_ (Figure 4G). GSH contents were decreased significantly with 3.9-fold under 36 mM H_2_O_2_ (Figure 4H). The contents of GSSG were increased 1.1-fold under 12 mM H_2_O_2_ and 1.2-fold under 24 mM H_2_O_2_, but decreased 1.2-fold under 36 mM H_2_O_2_ treatment (Figure 4H).

### 2.4. Identification of H_2_O_2_-Responsive Proteins in Leaves

**Figure 5:**
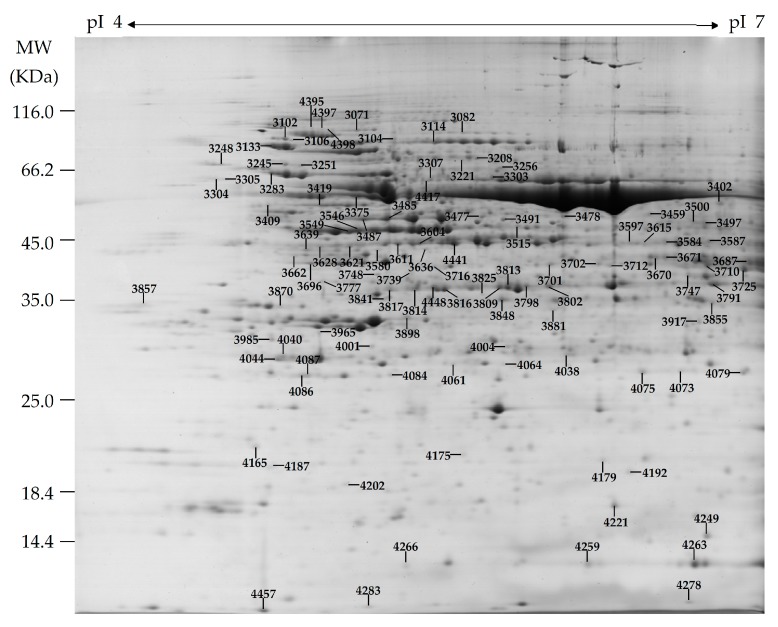
A representative 2DE gel images of proteins from leaves of *Populus simonii × Populus nigra*. Proteins were separated on 24 cm linear gradient immobilized pH gradient (IPG) strips (pH 4–7) using isoelectric focusing (IEF) in the first dimension, followed by 12.5% sodium dodecyl sulfate polyacrylamide gel electrophoresis (SDS-PAGE) gels in the second dimension. The 2DE gel was stained with Coomassie Brilliant Blue. Molecular weight (MW) in kilodaltons (KDa) and isoelectric point (pI) of proteins are indicated on the left and top of the gel, respectively. Eighty-one H_2_O_2_-responsive proteins identified by matrix-assisted laser desorption/ ionization (MALDI) tandem time of flight (TOF-TOF) mass spectrometry were marked with numbers on the gel, and the detailed information can be found in Table 1, Figure S1, and Table S1.

To explore the differential accumulated proteins (DAP) in *P. simonii × P. nigra* leaves in response to H_2_O_2_, the protein profiles in 0, 12, 24 and 36 mM H_2_O_2_-treated leaves were obtained using 2DE. On Coomassie Brilliant Blue (CBB)-stained gels (24 cm, pH 4–7 linear gradient immobilized pH gradient strips), 877 ± 21, 838 ± 23, 768 ± 48, and 811 ± 31 protein spots from 0, 12, 24 and 36 mM H_2_O_2_-treated leaves were detected, respectively (Figure 5 and Figure S1). Among them, 114 protein spots showed differential abundances in leaves under four distinct H_2_O_2_ concentrations (fold change > 1.5 and *p* < 0.05). All of the 114 DAP spots were subjected to in-gel digestion and protein identification using tandem mass spectrometry. A total of 83 DAPs were identified using matrix-assisted laser desorption/ ionization (MALDI) tandem time of flight (TOF-TOF) mass spectrometry (MS) and Mascot Searching with stringent criteria. Among the 83 identified DAP spots, 81 DAP spots contained a single protein each (Table 1 and Table S1), and the remaining two DAP spots contained more than one protein each (Table S2). Thus, the 81 DAPs were taken as H_2_O_2_-responsive proteins in leaves of *P. simonii × P. nigra*.

### 2.5. Annotation and Functional Categorization of the H_2_O_2_-Responsive Proteins

**Figure 6:**
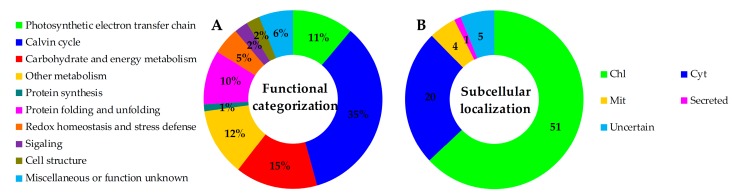
Functional categorization and subcellular localization of the identified 81 H_2_O_2_-responsive proteins from leaves of *Populus simonii × Populus nigra*. (**A**) The functional categories: The percentage of proteins in different functional categories is shown in the pie; (**B**) Subcellular localization groups of the identified proteins: The numbers of proteins with different locations are shown. Chl, chloroplast; Cyt, cytoplasm; Mit, mitochondrion.

Among the 81 H_2_O_2_-responsive proteins, 34 proteins were originally annotated as unknown, hypothetical proteins, or without annotation. They were all re-annotated according to the Basic Local Alignment Search Tool (BLAST) analysis (Table 1 and Table S3). Based on BLAST alignments, Gene Ontology, subcellular localization prediction, and information from literature, the 81 proteins were classified into ten functional categories including photosynthetic electron transfer chain, Calvin cycle, carbohydrate and energy metabolism, other metabolism, protein synthesis, protein folding and unfolding, redox homeostasis and stress defense, signaling, cell structure, and miscellaneous or function unknown (Table 1 and Figure 6A). Interestingly, proteins involved in photosynthetic electron transfer and Calvin cycle accounted for the largest group (46% of H_2_O_2_-responsive proteins). Besides, carbohydrate and energy metabolism (15%), other metabolism (12%), as well as protein folding and unfolding (10%) were also over-represented.

### 2.6. Subcellular Localization and Hierarchical Clustering of H_2_O_2_-Responsive Proteins

The subcellular localization of the 81 proteins was predicted using five different tools (i.e., YLoc, LocTree3, Plant-mPLoc, ngLOC, and TargetP) (Figure 6B, Table 1 and Table S4). In total, 51 proteins (63%) were predicted to be localized in chloroplasts, 20 in cytoplasm, four in mitochondria, one secreted, and five uncertain. This implied that most chloroplast proteins were obviously affected by H_2_O_2._

### 2.7. Hierarchical Clustering and Analysis of H_2_O_2_-Responsive Proteins

**Figure 7:**
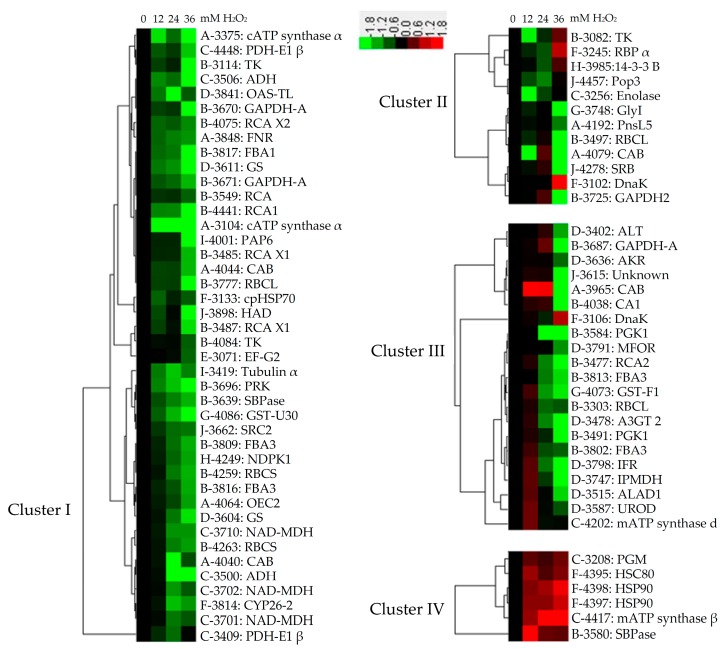
Hierarchical clustering analysis of 81 H_2_O_2_-responsive proteins in leaves of *Populus simonii × Populus nigra*. The four columns represent different treatments, including 0, 12, 24 and 36 mM H_2_O_2_. The rows represent individual proteins. The increased or decreased proteins are indicated in red or green, respectively. The color intensity increases with increasing abundant differences, as shown in the scale bar. The scale bar indicates log (base2) transformed protein abundance ratios ranging from −1.8 to 1.8. Functional categories indicated by capital letters, spot numbers, and protein names are listed on the right side. A, photosynthetic electron transfer chain; B, Calvin cycle; C, carbohydrate and energy metabolism; D, other metabolisms; E, protein synthesis; F, protein folding and unfolding; G, redox homeostasis and stress defense; H, signaling; I, cell structure; J, miscellaneous or function unknown. The abbreviations refer to Table 1.

To better understand the abundance patterns of the coordinately regulated proteins, hierarchical clustering analysis of the 81 H_2_O_2_-responsive proteins were performed, which revealed four main clusters (Figure 7). Cluster I included a total of 42 proteins, as the most group cluster, which included the significantly decreased proteins under H_2_O_2_ treatment. Cluster II included the proteins decreased under 12 mM, but increased under 24 or 36 mM H_2_O_2_ treatment. Cluster III contained the proteins unchanged or increased under relative lower concentration of H_2_O_2_ stress, but decreased under relative higher concentration, especially 36 mM H_2_O_2_ treatment. Several proteins involved in carbohydrate and energy metabolism were grouped into this subcluster. Cluster IV contained six proteins whose abundances were increased under H_2_O_2_ treatment. Notably, several heat shock proteins (HSPs) were categorized into Cluster IV.

### 2.8. Protein–Protein Interaction (PPI) among H_2_O_2_-Responsive Proteins

**Figure 8:**
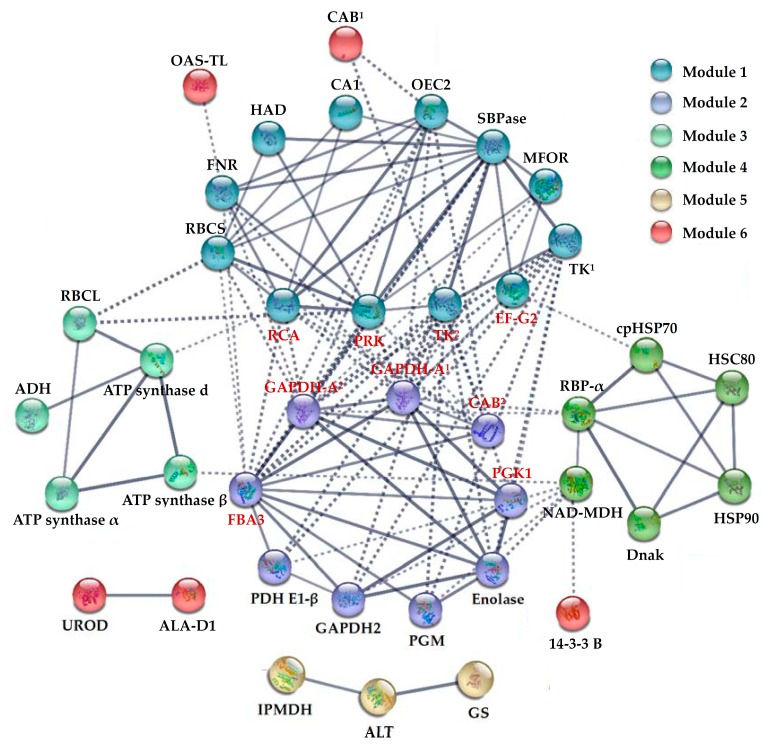
The protein–protein interaction (PPI) network in *Populus simonii × Populus nigra* leaves revealed by STRING analysis. A total of 81 H_2_O_2_-responsive proteins represented by 59 unique homologous proteins from Arabidopsis are shown in PPI network. Six main groups are indicated in different colors. The PPI network is shown in the confidence view generated by STRING database. Stronger associations are represented by thicker lines. The abbreviations refer to Table 1.

To discover the relationship of the 81 H_2_O_2_-responsive proteins, the PPI networks were generated using the web-tool STRING 10 [30]. Among the 81 H_2_O_2_-responsive proteins, 59 unique homologous proteins were found in Arabidopsis (Table S5) [31]. Out of the 59 proteins, 40 proteins were depicted in the STRING database (Figure 8). Six modules forming tightly connected clusters were illuminated, and stronger associations were represented by thicker lines in the networks (Figure 8). Twelve and nine proteins were connected in Module 1 and Module 2, respectively. Most of them were involved in photosynthesis or carbohydrate metabolism. Module 3 contained five proteins mainly involved in energy metabolism, and Module 4 contained six proteins mainly involved in protein folding. Three proteins involved in amino acid metabolism were assigned in Module 5.

## 3. Discussion

### 3.1. H_2_O_2_-Responsive Signal Transduction and Cellular Structure Modulation in Poplar

**Figure 9:**
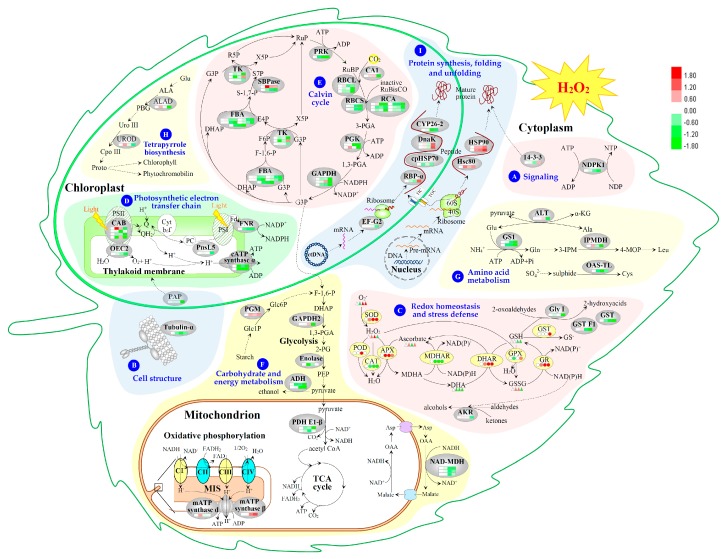
Schematic presentation of H_2_O_2_-responsive mechanism in leaves of *Populus simonii* × *Populus nigra*. The identified proteins were integrated into subcellular pathways: (**A**) signaling; (**B**) cell structure; (**C**), redox homeostasis and stress defense; (**D**) photosynthetic electron transfer chain; (**E**) calvin cycle; (**F**) carbohydrate and energy metabolism; (**G**) amino acid metabolism; (**H**) tetrapyrrole biosynthesis; and (**I**) protein synthesis, folding and unfolding. The abundances of identified proteins (shaded in gray ovals), enzyme activities (shaded in yellow ovals), and substrate contents are marked with squares, circles and triangles in different colors, respectively. The increased and decreased proteins, enzyme activities, and substrate contents are represented in red and green, respectively. The color intensity increases with increasing differences. The solid line indicates single-step reaction, the dashed line indicates multistep reaction, and the dotted line indicates the movement of proteins or substances. The abbreviations of identified proteins refer to Table 1. The abbreviations of metabolites: 1,3-PGA, 1,3-bisphosphoglycerate; 2-PG, 2-phosphoglycerate; 3-IPM, 3-isopropylmalate; 3-PGA, 3-phosphoglycerate; 4-MOP, 4-methyl-2-oxopentanoate; ADP, adenosine diphosphate; ALA, 5-aminolevulinicacid; Ala, alanine; Asp, aspartate; ATP, adenosine-triphosphate; CpoIII, coproporphyrinogen III; ctDNA, chloroplast DNA; Cys, cysteine; DHA, dehydroascrobate; DHAP, dihydroxyacetone phosphate; E4P, erythrose-4-phosphate; F-1,6-P, fructose-1,6-bisphosphate; F6P, fructose-6-phosphate; FADH_2_, reduced flavin adenine dinucleotide; Fd, ferredoxin; G3P, glyceraldehyde-3-phosphate; Glc1P, glucose-1-phosphate; Glc6P, glucose-6-phosphate; Gln, glutamine; Glu, glutamate; GSH, reduced glutathione; GSSG, oxidized glutathione; Leu, leucine; MDHA, monodehydroascrobate; MIS, mitochondrial intermembrane space; NAD^+^/NADH, nicotinamide adenine dinucleotide; NADP^+^/NADPH, nicotinamide adenine dinucleotide phosphate; NDP, nucleoside diphosphate; NTP, nucleoside triphosphate; OAA, oxaloacetic acid; PBG, porphobilinogen; PC, plastocyanin; PEP, phosphoenolpyruvate; pre-mRNA, precursor mRNA; Proto, protoporphyrin IX; PSI, photosystem I; PSII, photosystem II; Q, quinone; R5P, ribulose-5-phosphate; RuBP, ribulose-1,5-bisphosphate; RuP, ribulose-5-phosphate; S-1,7-P, sedoheptulose-1,7-bisphosphate; S7P, sedoheptulose-7-phosphate; TIC, translocon at the inner envelope membrane of chloroplasts; TOC, translocon at the outer envelope membrane of chloroplasts; UroIII, uroporphyrinogen III; X5P, xylulose-5-phosphate; α-KG, α-ketoglutarate.

H_2_O_2_ is generally regarded as a signal molecule in various abiotic/biotic stress-responsive pathways [6,7,8,9,10,11,12]. In this study, two signaling transduction-related proteins, 14-3-3 like protein B and nucleoside diphosphate kinase 1 (NDPK1), were altered in the H_2_O_2_-treated leaves of *P. simonii × P. nigra*. 14-3-3 like protein B was increased remarkably under 36 mM H_2_O_2_ (Table 1 and Figure 9A). Similarly, 14-3-3 like protein GF14-D was accumulated in *B. distachyon* leaves under 20 mM H_2_O_2_ for 4 h [19]. Plant 14-3-3 family proteins mediate the regulation of distinct biological processes by binding to phosphorylated client proteins. Recent proteome-wide in vivo approaches indicated that 14-3-3 proteins might interact with more than 100 target proteins in Arabidopsis [32]. Especially, plant 14-3-3 family was confirmed to be responsive to various environmental stresses [33]. In addition, NDPKs are multifunctional proteins that regulate a variety of eukaryotic cellular activities [34]. We found that NDPK1 was reduced in poplar leaves under H_2_O_2_ stress (Table 1 and Figure 9A), which was also reduced in Arabidopsis leaves in response to 3 mM H_2_O_2_ for five days [20]. However, H_2_O_2_ stress strongly induced the expression of *AtNDPK2* gene in Arabidopsis, which was associated with H_2_O_2_-mediated mitogen-activated protein kinase signaling in plants [34]. The different H_2_O_2_-responsive patterns between NDPK1 and NDPK2 may be attributed to the different functions of various plant NDPK types [35]. For instance, cytoplasm-localized Type I NDPKs are involved in growth, metabolism and stress responses, whereas chloroplast-localized Type II NDPKs are involved in photosynthetic development and oxidative stress management [35]. The specific functions of the NDPK family members need to be investigated in polar in response to H_2_O_2_.

Excessive H_2_O_2_ can damage protein and lipid structure, leading to the destruction of cell membrane stability. In this study, the increases of MDA content and REL implied that H_2_O_2_ induced plasma membrane lipid peroxidation and plasma membrane permeability in leaves of poplar (Figure 3A,B). Importantly, our proteomics results indicated that plastid lipid associated proteins (PAPs) and tubulin were 36 mM H_2_O_2_-reduced in leaves (Figure 9B). PAPs, also termed as fibrillin/CDSP34 proteins, are involved in the structural stabilization of thylakoid membrane upon environmental constraints. The gene expression and protein accumulation of PAPs were induced in tomato (*Lycopersicon esculentum*) and potato (*Solanum tuberosum*) under osmotic and oxidative stresses [36,37]. Besides, the expression of PAPs in *Brassica napus* and Arabidopsis were differentially regulated by various abiotic stresses, such as drought, ozone, cold, NaCl, light, and mechanical wounding [38,39]. The α/β-tubulin heterodimer is the building block of microtubules, which regulates cell division and expansion, as well as organelle movement. Microtubule organization and dynamics quickly responds to various external stress signals, such as low temperature [40], cold acclimation [41], as well as osmotic and salt stresses [42,43]. All these imply that cell structure modulation is critical for the tolerance of various exogenous stresses-induced intracellular oxidative stress in poplar.

### 3.2. H_2_O_2_-Induced Alteration of ROS Scavenging Pathways in Poplar

Generally, when leaves are exposed to H_2_O_2_ treatment, exogenous H_2_O_2_ can permeate through cell membrane into cells, leading to the increase of intracellular H_2_O_2_ level [17]. In this study, the H_2_O_2_ concentrations in leaves were increased under 12 and 24 mM H_2_O_2_ (Figure 4A). Interesting, the intracellular H_2_O_2_ levels were increased in leaves of grass pea (*Lathyrus sativu* L.) under 5 and 10 mM H_2_O_2_ for 24 h, but returned to the normal level under 20 mM H_2_O_2_, which were revealed from H_2_O_2_ content detection and the histochemical localization using DAB staining [44]. These implied that special pathways were employed for intracellular H_2_O_2_ scavenging when plants were exposed to relatively higher concentration of H_2_O_2_.

In poplar leaves, we found diverse antioxidative enzymes and antioxidants were involved in intracellular H_2_O_2_ scavenging to cope with exogenous H_2_O_2_ stress (Figure 9C). The increase of SOD activity would contribute to dismutase intracellular O_2_^•−^ to H_2_O_2_ in leaves under 24 and 36 mM H_2_O_2_ stress (Figure 4B). Interestingly, other primary antioxidative enzymes showed distinct activity patterns in response to various H_2_O_2_ concentrations. The activities of APX, POD, and GPX were induced under 12, 24 and 36 mM H_2_O_2_, respectively, indicating that different antioxidative pathways were employed under certain H_2_O_2_ level (Figure 4C,D). Similar results were also obtained in H_2_O_2_-stressed grass pea (*Lathyrus sativus* L.). The activities of APX and POD were increased under 5 and 10 mM H_2_O_2_, but returned to the normal level under 20 mM H_2_O_2_ [44]. Importantly, in this study, the induced GPX activity would especially facilitate to reduce relative higher intracellular H_2_O_2_ levels (Figure 4D), and enhance the reduction of lipid peroxide for defensing against oxidative membrane damage in poplar leaves under 36 mM H_2_O_2_ stress [45]. Unexpectedly, the H_2_O_2_-reduced CAT activity implied that the intracellular H_2_O_2_ would not be mainly scavenged in peroxisome when poplar leaves expose to extracellular H_2_O_2_ stress (Figure 4B) [1].

The activities of several antioxidative enzymes (e.g., MDHAR, DHAR, and GR) were H_2_O_2_-modulated for regeneration of reduced AsA and GSH, such as reduced MDHAR activity, and increased activities of DHAR and GR in poplar (Figure 4E,F). MDHAR and DHAR catalyze the regeneration of AsA, using nicotinamide adenine dinucleotide phosphate (NADPH) and GSH as electron source/donor, respectively, while GR maintains the cellular reduced GSH pool through converting GSSG to GSH with NADPH. The stress-induced activities of DHAR and GR also have been reported in several plants (e.g., maize and pepper) in response to abiotic stresses, such as salinity, drought, low temperature, and heavy metal [46]. This suggests that the H_2_O_2_-induced activities of DHAR and GR would maintain the reduced AsA and GSH pools for antioxidative processes in leaves. In addition, the altered H_2_O_2_-reponsive contents of antioxidants AsA/DHA and GSH/GSSG indicated that they contributed to H_2_O_2_ scavenging, which would function as the substances of aforementioned antioxidative enzyme (i.e., APX, POD, GPX, and DHAR) systems, but also could directly reduce H_2_O_2_ as reductants in poplar leaves. Additionally, the exogenous H_2_O_2_-induced osmolytes (i.e., proline, soluble sugar, and glycine betaine) were suggested to protect cellular components from degeneration by scavenging ROS in poplar leaves (Figure 3C–E). Similarly, the accumulation of proline and soluble sugars was found in wheat leaves under H_2_O_2_ treatment [20].

### 3.3. H_2_O_2_-Altered Redox Homeostasis in Poplar Leaves

In addition to the ROS scavenging pathways, glutathione-S-transferase (GST), glyoxylase (Gly) and aldo/keto reductase (AKR) were altered in regulating secondary release of metabolite signals in poplar leaves to cope with H_2_O_2_ stress. GSTs are mostly known as detoxifiers of electrophilic compounds by covalently linking glutathione to hydrophobic substrates for sequestration or removal, which plays an important role in improving plant stress tolerance [47]. In this study, GST activity was increased significantly under 36 mM H_2_O_2_, but the abundances of GST U30 and GST F1 were decreased (Figure 4F and Figure 9C, and Table 1). It can be explained that the enzyme activity is determined by not only protein abundance, but also its changes in conformation and post-translational modification (PTM). Moreover, plant GST group is a large protein family containing at least eight classes, and each family member has different role in response to various stress conditions. For example, the abundances of *B. distachyon* GST1-like [19] and citrus GST [18] were H_2_O_2_-decreased in leaves, but the abundances of wheat GST 19E50 [20] and rice GST F11 [17] were H_2_O_2_-increased in leaves. Therefore, in this study, the reduced abundances of two GST members would not account for the overall GST activity in polar leaves to cope with H_2_O_2_. The PTM mechanisms of GSTs are valuable to be further investigated.

Gly system comprising of Gly I and Gly II is the primary route for detoxification of methylglyoxal that is a toxic byproduct inhibiting cell proliferation, protein degradation, and antioxidant defense system [48]. Gly I was accumulated in rice leaves under 0.6 and 3 mM H_2_O_2_ stresses [17], but decreased in citrus leaves under 10 mM for 8 h [18]. In addition, AKRs are involved in detoxifying lipid peroxidation derived reactive aldehydes, leading to enhance the tolerance against abiotic stress-induced oxidative stress [49]. In this study, all the abundances of Gly I and AKR maintained at normal levels under 12 and 24 mM H_2_O_2_, but decreased significantly under 36 mM H_2_O_2_ (Table 1 and Figure 9C). This implied that Gly and AKR systems were probably employed for detoxification under relative lower concentration of H_2_O_2_ stress.

### 3.4. H_2_O_2_-Reduced Photosynthesis in Poplar

Photosynthesis is sensitive to ROS accumulation resulted from diverse stresses, because most photosynthetic enzymes are the preferential targets for the oxidation. In this study, the H_2_O_2_ immersion resulted in the decreases of net photosynthesis and stomatal conductance of *P. simonii × P. nigra* seedlings (Figure 2A,B). Importantly, we found 37 H_2_O_2_-responsive proteins were involved in photosynthesis, which accounted for 46% of all the H_2_O_2_-responsive proteins in poplar leaves (Table 1, and Figure 6A and Figure 9D,E). This is similar to what happened in leaves from rice under 0.6 and 15 mM H_2_O_2_ for 6 h and citrus under 10 mM H_2_O_2_ for 8 h, respectively [17,18]. In these two studies, 32% and 28% H_2_O_2_-responsive proteins in rice and citrus were identified using 2DE-based proteomics approaches, respectively [17,18]. These proteins are involved in light harvest, oxygen evolving, electron transfer, ATP synthesis, and Calvin cycle. Most of them were decreased under certain H_2_O_2_ concentration, leading to photosynthesis decline in poplar (Figure 2A and Figure 9D,E, and Table 1). Interestingly, most of these proteins, except for PnsL5, were changed in H_2_O_2_-treated leaves from other trees (e.g., citrus [18]) and gramineous plants (e.g., rice [17], wheat [20], and *B. distachyon* [19]).

In this study, our 2DE-based proteomics investigation revealed that ten of the H_2_O_2_-resposive photosynthetic proteins had multi-proteoforms in poplar leaves in response to H_2_O_2_ (Table 1 and Figure 9D,E). These proteoforms were mainly resulted from various H_2_O_2_-induced PTMs, including oxidative modification. All these proteins have been found to be oxidized in Arabidopsis in response to H_2_O_2_ treatments using redox proteomics approaches [50,51,52,53]. This is consistent with the findings from citrus and Arabidopsis that most photosynthesis-related proteins were easily carbonylated [18] and/or oxidized [51] under H_2_O_2_ exposure. Therefore, it is necessary to investigate the protein redox PTMs using redox proteomics technologies.

### 3.5. H_2_O_2_-Responsive Carbohydrate and Other Metabolisms in Poplar

Carbohydrate and energy supply are essential for plants in response to oxidative stress [20]. Our proteomics results revealed that phosphoglucomutase (PGM), cytosolic GAPDH, and enolase were altered in poplar leaves under H_2_O_2_ stress (Figure 9F). The increase of cytosolic PGM would enhance reversible interconversion of glucose-1-phosphate and glucose-6-phosphate, providing substrates for glycolysis and synthesis of a variety of cellular constituents. In roots of two black poplar (*P. nigra*) clones, the PGM gene and soluble sugar level were all induced under drought stress [54]. This implied that the mobilization of stored starch would be triggered in poplar when carbon assimilation was inhibited due to oxidization stress-reduced photosynthesis [54]. Besides, the H_2_O_2_-decreased abundances of cytosolic GAPDH and enolase implied that glycolysis was inhibited in poplar under H_2_O_2_ stress. In addition, the decreased abundances of alcohol dehydrogenase and cytosolic NAD-dependent malate dehydrogenase would reduce the regeneration of reducing power nicotinamide adenine dinucleotide (NADH) in H_2_O_2_-stressed poplar leaves [55,56], while the abundance-altered pyruvate dehydrogenase E1 and mitochondria ATP synthase would contribute for modulation of tricarboxylic acid cycle and energy supply in H_2_O_2_-stressed poplar leaves.

Glutamate and cysteine are central metabolites that serve as donors for the synthesis of other amino acids, vitamins, coenzymes, GSHs, and proteins, which play critical roles in plant stress responses. The syntheses of glutamine/glutamate and cysteine are depended on glutamine synthetase (GS)-involved pathway [57] and *O*-acetylserine (thiol) lyase (OAS-TL)-involved pathway [58], respectively. In this study, two proteoforms of cytosolic GS and OAS-TL were decreased in poplar leaves under H_2_O_2_ stress (Table 1, Figure 9G). The oxidative-decreased GS was also found in wheat leaves under 15 mM H_2_O_2_ for five days [20]. In addition, two other amino acid metabolism-related enzymes (i.e., 3-isopropyl malate dehydrogenase and alanine aminotransferase family protein) were decreased in poplar leaves under 36 mM H_2_O_2_ stress (Table 1 and Figure 9G). These proteomics results implied that amino acid metabolism was reduced in poplar leaves under H_2_O_2_ stress.

In addition, two enzymes in plant tetrapyrrole biosynthetic pathway, delta-aminolevulinic acid dehydratase 1 (ALAD1) and uroporphyrinogen decarboxylase (UROD), were all decreased under 36 mM H_2_O_2_ stress (Table 1, Figure 9H). ALAD catalyzes the asymmetric condensation of two molecules of δ-aminolevulinic acid to porphobilinogen, and UROD catalyzes the formation of coproporphyrinogen from uroporphyrinogen. Altered ALAD activity concomitant with reduced chlorophyll content have been reported in many terrestrial plants exposed to various metal (e.g., aluminum, cadmium, and lead) stresses [59,60,61]. Interestingly, transgenic tobacco plants with reduced activity of UROD was characterized by the accumulation of photosensitizing tetrapyrrole intermediates, which would induce the enzymatic detoxifying defense system, and especially resemble the hypersensitive reaction observed after pathogen attack [62]. Thus, the reduction of ALAD1 and UROD might result in the accumulation of photosensitizing tetrapyrrole intermediates, which probably play roles in the response of H_2_O_2_.

### 3.6. H_2_O_2_-Responsive Proteins Conformation in Poplar

Maintaining proteins in their functional conformations and preventing the aggregation of non-native proteins are important for plant survival under oxidative conditions. HSPs and other chaperones are responsible for protein folding, assembly, translocation, and degradation in response to stress conditions [63]. In this study, a RuBisCO large subunit-binding protein α subunit (RBP-α) and two proteoforms of chaperone DnaK (DnaK) were increased remarkably in poplar under 36 mM H_2_O_2_ stress (Table 1 and Figure 9I). RBP is considered as chloroplast chaperonin 60 (Cpn60), which is most likely involved in mature protein folding/assembly in plants, and facilitates the translocated protein to fold into native conformation. Previous studies showed that RBPs were induced in wheat leaves under drought [64] and salt stress [65], as well as in rice leaves by H_2_O_2_ stress [17]. Moreover, Cpn60 subunit β can protect RuBisCO activase from thermal denaturation and function in acclimating photosynthesis to heat stress [66]. In this study, the two proteoforms of DnaK in poplar have relatively high sequence similarity to chloroplast HSPs [67]. The chloroplast HSPs carry out pivotal function in processes related to growth and development and in response to diverse environmental stresses, such as heat, light, and pathological stress [67]. For example, the expression of the chloroplast-localized Hsp70B is induced in *Chlamydomonas* under heat shock, high light and oxidative stresses [68]. A wheat chloroplast TaHsp70 plays a critical role in defense response elicited by infection of stripe rust fungus [69]. Therefore, the increases of RBP-α and DnaKs in poplar under 36 mM H_2_O_2_ treatment suggest they play an important role in protection against the high dose oxidative stress.

In addition, heat shock cognate protein 80 (HSC80) and two proteoforms of heat shock protein 90 (HSP90) were increased in poplar under H_2_O_2_ stress (Table 1, Figure 9I). HSC80 was found to be increased 10-fold in tomato cell culture upon heat shock [70]. HSP90 is distinct from many other molecular chaperones in that most of its known substrates to date are signal transduction-related proteins such as steroid hormone receptors and signaling kinases [63]. Recent studies revealed that plant HSP90s were important in plant development, environmental stress response, as well as disease and pest resistance [71]. Therefore, the induced HSC80 and HSP90s might prevent the aggregation of non-native proteins and reestablish normal protein conformation in H_2_O_2_-stressed poplar leaves.

## 4. Methods

### 4.1. Plant Cultivation and Treatment

The terminal buds or lateral buds excised from *P. simonii × P. nigra* plantlets were transferred to a culture flask containing 80 mL 1/2 MS solid medium, containing 2% (*w*/*v*) sugar and 0.54% (*w*/*v*) agar. The explants were cultured in a phytotron at 26 °C/22 °C (day/night), 16 h photoperiod and 200 μmol·m^−2^·s^−1^ light intensity for 50 days. The shoots of regenerated plantlets were immersed in 0, 12, 24 and 36 mM H_2_O_2_ for 6 h, respectively. After the treatments, leaves were harvested and blotted dry on filter paper immediately. For each treatment, at least three biological replicates were performed. For each replicate, more than three whole leaves with similar size from at least three separate poplar seedlings were collected and pooled. The fresh weight was 0.2 g. The samples were either used fresh or stored at −80 °C for future analysis.

### 4.2. Photosynthesis Measurement

Pn, Gs, Ci, and Tr were measured in fully expanded leaves of each plant using a portable photosynthesis system LICOR 6400 XT (LI-COR Inc., Lincoln, NE, USA) [72]. The measurements were done at 10:00 a.m. At least nine leaves for each sample were measured.

### 4.3. Determination of MDA Content, REL, Total Soluble Sugar, Proline, and Glycine Betaine Contents

The MDA content and REL were determined using previous methods described by Wang et al. [73]. For the MDA content assay, 0.2 g fresh leaves were ground in 5 mL 10% trichloroacetic acid, and centrifuged at 10,000× *g* at 4 °C for 10 min, then the supernatant was collected. Two milliliters of 0.6% (*w*/*v*) thiobarbituric acid solution was added to 2 mL of the supernatant. The reaction solution was incubated for 15 min at 100 °C followed by cooling down to ambient temperature. The absorbance was detected under 450, 532 and 600 nm using an Ultrospec 2100 pro UV/Visible spectrophotometer (GE Healthcare Life Science, Uppsala, Sweden). The MDA content was calculated according to Li et al. [74].

For the REL determination, 0.2 g fresh leaves were cut and completely immersed in 20 mL deionized water, then degassed for 10 min. The electrical conductivity of the solution (E1) was measured using a conductivity instrument (DDS-11A) after 20 min. Subsequently, the solution was incubated at 100 °C for 15 min and cooled to room temperature, and then the electrical conductivity of the solution (E2) was determined. In addition, the electrical conductivity of deionized water (E0) was also detected. The REL was calculated according to the equation: REL (%) = (E1 − E0)/(E2 − E0) × 100%.

Contents of proline and total soluble sugar were determined using ninhydrin reaction and an anthrone reagent method developed by Li et al. [74]. For the proline determination, 0.2 g fresh leaves were ground in 3 mL 3% (*w*/*v*) sulfosalicylic acid. After incubation at 100 °C for an hour and followed by cooling down to ambient temperature, the homogenate was centrifuged at 15,000× *g* for 10 min at 25 °C. Then, 1 mL supernatant, 2 mL glacial acetic acid, and 2 mL ninhydrin were incubated at 100 °C for an hour. After cooled to room temperature, the incubated solution with addition of 8 mL methylbenzene was allowed to stand for an hour before the detection at 520 nm using a spectrophotometer.

For the total soluble sugar assay, 0.2 g fresh leaves were ground in 5 mL deionized water. The homogenate was incubated at 100 °C for 30 min followed by cooling down to ambient temperature. After centrifugation at 15,000× *g* for 10 min at 25 °C, the supernatant was collected and diluted with deionized water to 50 mL. Then, 1 mL the extracting solution, 0.5 mL 2% (*w*/*v*) ethyl acetate solution of anthrone, and 5 mL concentrated sulfuric acid were incubated at 100 °C for 2 min. The absorbance was detected under 630 nm using a spectrophotometer after cooled down to room temperature.

Glycine betaine content was measured using reinecke salt method as described by Zhao et al. [75]. Fresh leaves (0.2 g) were ground in liquid nitrogen, and then the powder was incubated for 24 h in 6 mL 0.375% (*w*/*v*) reinecke salt solution. The homogenate was centrifuged at 10,000× *g* for 15 min at 20 °C. The supernatant was collected and filtered through a 0.45-μm-pore-size cellulose acetate filter. The filtrate was dried at 70 °C and then resuspended in 5 mL deionized water. After 5 mL reinecke salt solution was added, the reaction solution was incubated at 4 °C for 2 h, and then centrifuged at 4000× *g* for 15 min at 4 °C. The precipitate was collected, and then redissolved in 15 mL aether. Then, the solution was centrifuged at 4000× *g* for 15 min at 4 °C. The supernatant was harvested, dried, and redissolved in 70% acetone. The absorbance was determined at 525 nm using a spectrophotometer. The contents of proline, total soluble sugar, and glycine betaine content were calculated from the standard curve.

### 4.4. Determination of ROS and Antioxidant Substances Contents, and Antioxidant Enzyme Activity Assay

The content of H_2_O_2_ was determined using potassium iodide reaction as described in Suo et al. [76]. Generation rate of O_2_^•−^ was obtained according to a method of Zhao et al. [75]. For antioxidant enzyme activity assay, 0.2 g leaves were homogenized on ice in 3 mL 50 mM phosphate buffer (pH 7.8, and containing 2% PVP-40 and 2 mM ascorbate). After centrifugation at 15,000× *g* for 20 min at 4 °C, the supernatant was collected for enzyme activity assays, including SOD, CAT, APX, GPX, POD, MDHAR, DHAR, GR, and GST. The activities of SOD, CAT, APX, POD, GR, and GST were assayed according to our previous methods [72]. The activities of GPX, MDHAR, and DHAR were measured according to our previous methods described by Suo et al. [76]. For all the enzyme activity assays, protein content was determined using the Bradford method [77]. In addition, contents of AsA, DHA, GSH, and GSSG were measured according to methods of Wei et al. [78].

### 4.5. Protein Sample Preparation, 2DE, and Protein Abundance Analysis

The proteins from leaves under different concentrations of H_2_O_2_ treatments were extracted using a phenol extraction method according to Wang et al. [73]. About 1.6 mg protein was loaded on per gel, separated on linear gradient IPG strips (24 cm, pH 4-7) through isoelectric focusing (IEF) in the first dimension, followed by 12.5% SDS-PAGE gels in the second dimension, and stained by CBB staining. Gel image acquisition and analysis were conducted as described in detail in Wang et al. [73]. For quantitative analysis, the volume of each spot was normalized against the total valid spots. The protein spots displaying consistent abundance changes from three biological replicates with greater than 1.5-fold changes and a *p* value smaller than 0.05 were considered to be DAP [72].

### 4.6. Protein Identification by MALDI-TOF/TOF MS and Database Searching

The DAP spots were excised from the gels and digested with trypsin as previously described [73]. MS/MS spectra were obtained using an ABI 5800 MALDI TOF/TOF MS (AB Sciex, Foster City, CA, USA). The mass error was below 30 ppm at both MS and MS/MS mode, and the resolution was 10,000. The MS/MS spectra were subjected to the online Mascot program [79] to search against all green plants (Viridiplantae) in NCBInr protein databases [80]. The searching parameters were set according to Wang et al. [73], the mass accuracy was 0.3 Da, and the maximum number of missed cleavages was set to one. To obtain high confident identification, proteins had to meet the following criteria: (1) the top hits on the database searching report; (2) a probability-based MOWSE score greater than 52 (*p <* 0.05); and (3) more than two peptides matched with nearly complete y-ion series and complementary b-ion series.

### 4.7. Protein Classification, Subcellular Localization, Hierarchical Cluster Analysis, and Protein–Protein Interaction Prediction

The identified proteins were searched against the NCBI database [80] and UniProt database [81] to determine if their functions were known. Combined with knowledge from BLAST alignments and literature, proteins were classified into different categories.

The subcellular localization of the identified proteins were predicted using five Internet tools according to Suo et al. [76]: YLoc [82], LocTree3 [83], Plant-mPLoc [84], ngLOC [85], and TargetP [86]. Only the consistent predictions meeting the high criteria from at least two tools were accepted as a confident result.

Self-organizing tree algorithm hierarchical clustering of the protein abundance profiles was obtained from log (base 2) transformed fold change values of protein spots using Cluster software (version 3.0).

The protein–protein interactions were predicted using the web tool STRING 10 [30]. The homologs of the DAPs in Arabidopsis were obtained by sequence BLAST in TAIR database [31], and then the homologs were subjected to the web tool of STRING 10 for creating functional protein association networks, based on published literature, genome analysis of domain fusion, gene neighborhood, phylogenetic profiling/homology, co-expression, co-occurrence, and other experimental evidence [76].

### 4.8. Statistical Analysis

All the physiological results were presented as means ± standard deviation (SD) of at least three biological replicates. Data were analyzed by one-way ANOVA followed by Duncan's test using the statistical software SPSS 17.0 (SPSS Inc., Chicago, IL, USA). A *p* value smaller than 0.05 was considered to be statistically significant.

## 5. Conclusion

In the course of poplar development and stress-response in poplars, the molecular regulation of H_2_O_2_ homeostasis in the cells is complicated and fine-tuned. In the present study, we present a primary H_2_O_2_-responsive network in leaves of poplar (*P. simonii × P. nigra*) using integrative analysis of physiological and proteomic approaches. The molecular network includes 14-3-3 protein-/NDPK-mediated signaling pathway, dynamics of thylakoid membrane structure, enhancement of diverse antioxidative defense system, alteration of photosynthesis, adjustment of carbohydrate and other basic metabolisms, as well as modulation of protein synthesis and conformation (Figure 9). This study provides new information and insights into underlying H_2_O_2_-responsive mechanisms in poplar plants. Although our proteomics results highlighted some critical candidate proteins/genes in H_2_O_2_-responsive signaling and metabolic pathways, their biological functions for H_2_O_2_ response in poplar still need future characterization using molecular genetics and PTM analysis tools.

## Figures and Tables

**Table 1:** H_2_O_2_-responsive proteins in leaves of *Populus simonii × Populus nigra*.

Spot No. ^(*a*)^	Protein Name ^(*b*)^	Abbreviation	Subcellular Location ^(*c*)^	Accession No. ^(*d*)^	Sco ^(*e*)^	QM ^(*f*)^	V%±SD ^(*g*)^ 0 12 24 36
**Photosynthetic electron transfer chain (9)**							
4044	Chlorophyll A/B binding protein	CAB	Chl	AAA18529	135	2	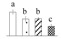
3965	Chlorophyll A/B binding protein	CAB	Chl	AAA18529	153	2	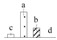
4040	Light harvesting chlorophyll A/B binding protein	CAB	Chl	ABW70800	213	3	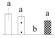
4079	Chlorophyll A-B binding family protein*	CAB	Chl	ABK96765	201	3	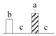
4064	Photosystem II oxygen-evolving complex protein 2 precursor	OEC2	Chl	XP_002300858	136	2	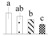
4192	Photosynthetic NDH subunit of luminal location 5, chloroplastic*	PnsL5	Chl	CDP13378	102	2	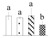
3848	Ferredoxin-NADP reductase, chloroplastic*	FNR	Chl	OAY57547	310	5	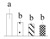
3375	ATP synthase CF1 α subunit, chloroplastic	cATP synthase α	Chl	AKF33906	369	6	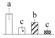
3104	ATP synthase CF1 α subunit, chloroplastic	cATP synthase α	Chl	AKF33906	164	2	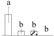
**Calvin cycle (28)**							
4038	Carbonic anhydrase isoform 1*	CA1	Chl	ABK96336	187	3	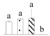
3549	Ribulose-bisphosphate carboxylase (RuBisCO) activase, chloroplastic	RCA	Chl	Q01587	316	4	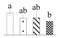
4441	RuBisCO activase 1, chloroplastic	RCA1	Chl	Q7X9A0	162	2	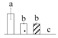
3485	RuBisCO activase, chloroplastic isoform X1*	RCA X1	Chl	ABK96359	543	6	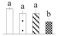
3487	RuBisCO activase, chloroplastic isoform X1*	RCA X1	Chl	ABK96359	401	6	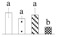
3477	Rubisco activase isoform 2*	RCA2	Chl	CDP00127	294	5	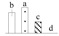
4075	RuBisCO activase, chloroplastic isoform X2	RCA X2	Chl	XP_011002635	294	5	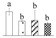
3777	RuBisCO large chain	RBCL	Chl	O78258	176	2	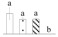
3303	RuBisCO large subunit	RBCL	Chl	CUR00003	176	2	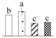
3497	RuBisCO large chain	RBCL	Chl	O78258	108	2	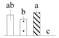
4259	RuBisCO small chain	RBCS	Chl	XP_011035062	243	6	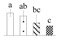
4263	RuBisCO small chain	RBCS	Chl	XP_002531624	112	2	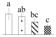
3584	Phosphoglycerate kinase 1 family protein	PGK1	Chl	XP_002315066	351	7	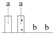
3491	Phosphoglycerate kinase 1 family protein	PGK1	Chl	XP_002315066	351	7	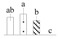
3687	Glyceraldehyde-3-phosphate dehydrogenase A, chloroplastic*	GAPDH-A	Chl	ABK94956	484	6	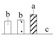
3671	Glyceraldehyde-3-phosphate dehydrogenase A, chloroplastic*	GAPDH-A	Chl	OIW03351	166	2	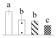
3670	Glyceraldehyde-3-phosphate dehydrogenase A, chloroplastic*	GAPDH-A	Chl	OIW03351	113	2	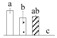
3817	Fructose-bisphosphate aldolase 1, chloroplastic*	FBA1	Chl	ABK95613	164	3	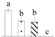
3813	Fructose-bisphosphate aldolase 3	FBA3	Chl	AGB05600	185	3	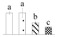
3816	Fructose-bisphosphate aldolase 3	FBA3	Chl	AGB05600	196	3	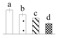
3809	Fructose-bisphosphate aldolase 3	FBA3	Chl	AGB05600	124	2	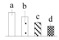
3802	Fructose-bisphosphate aldolase 3	FBA3	Chl	AGB05600	133	2	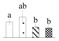
3082	Transketolase, chloroplastic	TK	Chl	KHG01555	99	2	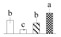
3114	Transketolase, chloroplastic	TK	Chl	EMT02862	120	2	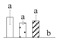
4084	Transketolase, chloroplastic	TK	Chl	Q43848	92	2	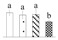
3639	Sedoheptulose-1,7-bisphosphatase, chloroplastic*	SBPase	Chl	XP_002316235	57	2	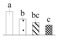
3580	Sedoheptulose-1,7-bisphosphatase, chloroplastic*	SBPase	Chl	OAY38482	101	3	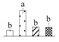
3696	Phosphoribulokinase, chloroplastic*	PRK	Chl	AOL56425	92	2	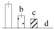
**Carbohydrate and energy metabolism (12)**							
3208	Phosphoglucomutase, cytoplasmic	PGM	Cyt	Q9ZSQ4	115	3	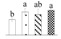
3725	Glyceraldehyde-3-phosphate dehydrogenase 2, cytosolic*	GAPDH2	Cyt	XP_002318114	374	4	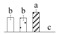
3256	Enolase	―	Cyt	Q42971	86	2	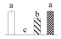
3506	Alcohol dehydrogenase*	ADH	Cyt	XP_002302195	133	5	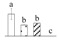
3500	Alcohol dehydrogenase*	ADH	Cyt	XP_002302195	171	3	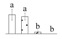
3701	NAD-dependent malate dehydrogenase	NAD-MDH	Cyt	AAL11502	161	2	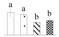
3702	NAD-dependent malate dehydrogenase	NAD-MDH	Cyt	AAL11502	104	2	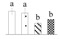
3710	NAD-dependent malate dehydrogenase	NAD-MDH	Cyt	AAL11502	223	3	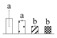
4448	Pyruvate dehydrogenase E1 component subunit beta, mitochondrial*	PDH E1-β	Mit	GAU16570	249	3	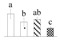
3409	Pyruvate dehydrogenase E1 component subunit beta, mitochondrial*	PDH E1-β	Mit	GAU16570	221	3	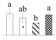
4202	ATP synthase subunit d, mitochondrial *	mATP synthase d	Mit	CBI31501	89	2	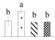
4417	ATP synthase subunit beta, mitochondrial *	mATP synthase β	Mit	CDP00716	66	2	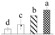
**Other metabolism (10)**							
3611	Glutamine synthetase	GS	Cyt	AGG19203	179	3	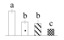
3604	Glutamine synthetase	GS	Cyt	ABF06665	150	3	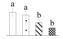
3841	O-acetylserine (thiol) lyase family protein	OAS-TL	Uncertain	XP_006389317	70	2	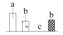
3747	3-isopropyl malate dehydrogenase, chloroplastic	IPMDH	Chl	P29696	117	2	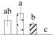
3402	Alanine aminotransferase family protein	ALT	Uncertain	ALT55639	239	6	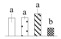
3515	Aldolase superfamily protein isoform 1, delta-aminolevulinic acid dehydratase 1, chloroplastic*	ALAD1	Chl	EOY16322	92	3	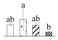
3587	Uroporphyrinogen decarboxylase	UROD	Chl	XP_011012304	272	4	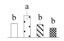
3478	Anthocyanidin 3-O-glucosyltransferase 2*	A3GT 2	Uncertain	ABK96136	212	3	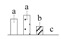
3798	Isoflavone reductase family protein*	IFR	Cyt	ABK95019	133	3	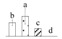
3791	2-methylene-furan-3-one reductase*	MFOR	Chl	ABK96279	238	3	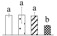
**Protein synthesis (1)**							
3071	Elongation factor G-2, chloroplastic*	EF-G2	Chl	XP_002304430	108	4	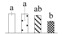
**Protein folding and unfolding (8)**							
3245	RuBisCO large subunit-binding protein subunit α	RBP-α	Chl	XP_011000529	99	3	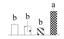
3133	Stromal 70 kDa heat shock-related family protein	cpHSP70	Chl	XP_006389517	606	7	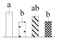
3102	Chaperone DnaK	DnaK	Chl	KVI03056	445	7	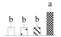
3106	Chaperone DnaK	DnaK	Chl	KVI03056	275	4	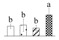
4395	Heat shock cognate protein 80	HSC80	Cyt	P36181	87	2	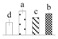
4397	Heat shock protein 90	HSP90	Cyt	KVI10442	74	2	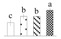
4398	Heat shock protein 90	HSP90	Cyt	KVI10442	71	2	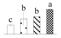
3814	Peptidyl-prolyl cis-trans isomerase CYP26-2, chloroplastic*	CYP26-2	Chl	XP_002318560	281	4	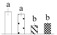
**Redox homeostasis and stress defense (4)**							
4086	Glutathione S-transferase U30	GST-U30	Cyt	ANO39995	74	2	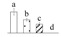
4073	Glutathione S-transferase F1	GST-F1	Cyt	ANO39924	164	2	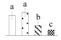
3748	Glyoxalase I homolog family protein	GlyI	Cyt	XP_002305564	88	2	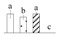
3636	Aldo/keto reductase family protein	AKR	Chl	XP_002302125	296	4	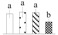
**Signaling (2)**							
4249	Nucleoside diphosphate kinase 1*	NDPK1	Cyt	ABK95604	139	25%	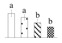
3985	14-3-3-like protein B*	14-3-3 B	Cyt	XP_002306545	119	12%	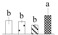
**Cell structure (2)**							
3419	Tubulin α chain	Tubulin-α	Cyt	Q9FT36	101	12%	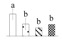
4001	Plastid-lipid-associated protein 6, chloroplastic*	PAP6	Chl	AAR26489	154	18%	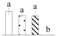
**Miscellaneous or function unknown (** **5)**							
3898	Haloacid dehalogenase-like hydrolase family protein*	HAD	Chl	ABK96272	114	7%	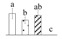
4457	Pop3 peptide family protein*	Pop3	Uncertain	1SI9_A	207	51%	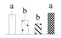
3662	Soybean genes regulated by cold 2-like domain*	SRC2	Uncertain	XP_011030162	136	10%	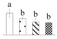
4278	Stress responsive A/B barrel domain*	SRB	Chl	CAA39082	132	24%	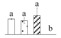
3615	Unknown protein	―	Secreted	ABK94923	93	7%	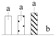

*^a^* Assigned spot number as indicated in Figure 5. *^b^* The name of the proteins identified by MALDI TOF/TOF MS. Protein names marked with an asterisk (*) have been edited based on BLAST against NCBI non-redundant protein database. The detailed information of the NCBI BLAST can be found in Table S2. *^c^* Protein subcellular localization predicted by software YLoc, LocTree3, Plant-mPLoc, ngLOC, and TargetP. Chl, chloroplast; Cyt, cytoplasm; Mit, mitochondrion. *^d^* Database accession numbers from NCBInr. *^e^* The Mascot score obtained after searching against the NCBInr database. *^f^* The number of unique peptides identified for each protein. *^g^* The mean values of protein spot volumes relative to total volume of all the spots. The different small letters on the columns indicate significant differences (*p* < 0.05) among the four samples as determined by one-way ANOVA. Error bars indicate ± SD.

## Supplementary Materials

Supplementary materials can be found at www.mdpi.com/1422-0067/18/10/2085/s1.

## Abbreviations

14-3-3 B	14-3-3-like protein B
2DE	Two-dimensional gel electrophoresis
^1^O_2_	Singlet oxygen
A3GT 2	Anthocyanidin 3-O-glucosyltransferase 2
ADH	Alcohol dehydrogenase
AKR	Aldo/keto reductase family protein
ALAD1	Delta-aminolevulinic acid dehydratase 1
ALT	Alanine aminotransferase family protein
APX	Ascorbate peroxidase
AsA	Ascorbate
BLAST	Basic local alignment search tool
CA1	Carbonic anhydrase isoform 1
CAB	Chlorophyll A/B binding protein
CAT	Catalase
cATP synthase α	ATP synthase CF1 α subunit
CBB	Coomassie brilliant blue
Ci	Intercellular CO2 concentration
cpHSP70	Stromal 70 kda heat shock-related family protein
CYP26-2	Peptidyl-prolyl cis-trans isomerase CYP26-2
DAP	Differentially accumulated protein
DHA	Dehydroascorbate
DHAR	Dehydroascorbate reductase
DnaK	Chaperone Dnak
EF-G2	Elongation factor G-2
ESTs	Expressed sequence tags
FBA	Fructose-bisphosphate aldolase
FNR	Ferredoxin-NADP reductase
GAPDH2	Glyceraldehyde-3-phosphate dehydrogenase 2
GAPDH-A	Glyceraldehyde-3-phosphate dehydrogenase A
Gly I	Glyoxalase I homolog family protein
GPX	Glutathione peroxidase
GR	Glutathione reductase
GS	Glutamine synthetase
Gs	Stomatal conductance
GSH	Reduced glutathione
GSSG	Oxidized glutathione
GST	Glutathione S-transferase
GST-F1	Glutathione S-transferase F1 (GST-F1)
GST-U30	Glutathione S-transferase U30
H_2_O_2_	Hydrogen peroxide
HAD	Haloacid dehalogenase-like hydrolase family protein
HO^•^	Hydroxyl radicals
HSC80	Heat shock cognate protein 80
HSP90	Heat shock protein 90
IFR	Isoflavone reductase family protein
IPG	Immobilized pH gradient
IPMDH	3-isopropyl malate dehydrogenase
iTRAQ	Isobaric tags for relative and absolute quantification
mATP synthase d	ATP synthase subunit d
mATP synthase β	ATP synthase subunit beta
MDA	Malondialdehyde
MDHAR	Monodehydroascorbate reductase
MFOR	2-methylene-furan-3-one reductase
MALDI	Matrix-assisted laser desorption/ ionization
MS	Mass spectrometry
NAD-MDH	NAD-dependent malate dehydrogenase
NADH	Nicotinamide adenine dinucleotide
NADPH	Nicotinamide adenine dinucleotide phosphate
NDPK1	Nucleoside diphosphate kinase 1
O_2_^•−^	Superoxide anion radicals
OAS-TL	O-acetylserine (thiol) lyase family protein
OEC2	Photosystem II oxygen-evolving complex protein 2 precursor
PAP6	Plastid-lipid-associated protein 6
PDH E1-β	Pyruvate dehydrogenase E1 component subunit beta
PGK1	Phosphoglycerate Kinase 1
PGM	Phosphoglucomutase
Pn	Net photosynthetic rate
PnsL5	Photosynthetic NDH subunit of lumenal location 5
POD	Peroxidase
Pop3	Pop3 peptide family protein
PRK	Phosphoribulokinase
PTM	post-translational modification
RBCL	Rubisco large chain
RBCS	Rubisco small chain
RBP-α	Rubisco large subunit-binding protein subunit α
RCA	Rubisco activase
REL	Relative electrolyte leakage
ROS	Reactive oxygen species
RuBisCO	Ribulose-bisphosphate carboxylase
SBPase	Sedoheptulose-1, 7-bisphosphatase
SOD	Superoxide dismutase
SRB	Stress responsive A/B Barrel domain
SRC2	Soybean genes regulated by cold 2 (SRC2)-like domain
TCA	Tricarboxylic acid
TK	Transketolase
TOF	Time of flight
Tr	Transpiration rate
Tubulin α	Tubulin α chain
UROD	Uroporphyrinogen decarboxylase
